# Wellens’ Syndrome from COVID-19 Infection Assessed by Enhanced Transthoracic Coronary Echo Doppler: A Case Report

**DOI:** 10.3390/diagnostics12040804

**Published:** 2022-03-25

**Authors:** Carlo Caiati, Paolo Desario, Giuseppe Tricarico, Fortunato Iacovelli, Paolo Pollice, Stefano Favale, Mario Erminio Lepera

**Affiliations:** Institute of Cardiovascular Disease, Department of Emergency and Organ Transplantations, University of Bari “Aldo Moro”, 70124 Bari, Italy; paolo.desario91@gmail.com (P.D.); gius931957@gmail.com (G.T.); fortunato.iacovelli@gmail.com (F.I.); paolo.pollice@yahoo.it (P.P.); stefano.favale@uniba.it (S.F.); marioerminio.lepera@uniba.it (M.E.L.)

**Keywords:** Wellens’ syndrome, acute coronary artery disease, enhanced transthoracic Doppler echocardiography, coronary blood flow, coronary flow reserve

## Abstract

Wellens’ syndrome (WS) is a preinfarction state caused by a sub-occlusion of the proximal left anterior descending coronary artery (LAD). In this case report, for the first time, we describe how this syndrome can be caused by COVID-19 infection and, most importantly, that it can be assessed bedside by enhanced transthoracic coronary echo Doppler (E-Doppler TTE). This seasoned technique allows blood flow Doppler to be recorded in the coronaries and at the stenosis site but has never been tested in an acute setting. Two weeks after clinical recovery from bronchitis allegedly caused by COVID-19 infection on the basis of epidemiologic criteria (no swab performed during the acute phase but only during recovery, at which time it was negative), our patient developed typical angina for the first time, mainly during effort but also at rest. He was admitted to our tertiary center, where pharyngeal swabs tested positive for COVID-19. A typical EKG finding supporting WS prompted an assessment of the left main coronary artery (LMCA) and the whole LAD blood flow velocity by E-Doppler TTE. Localized high velocity (transtenotic velocity) (100 cm/s) was recorded in the proximal LAD, with the reference velocity being 20 cm/s. This indicated severe stenosis with 90% area narrowing according to the continuity equation, as confirmed by coronary angiography. During follow-up after successful stenting, E-Doppler TTE showed a decrease in the transtenotic acceleration, confirming stent patency and a normal coronary flow reserve (3.2) and illustrating a normal microcirculatory function. Conclusion: COVID infection can trigger a coronary syndrome like WS. E-Doppler TTE, an ionizing radiation-free method, allows safe and rapid bedside management of the syndrome. This new strategy can be pivotal in distinguishing true WS from pseudo-WS. In cases of pseudo-WS, coronary angiography can be avoided. If E-Doppler TTE confirms the stenosis and PCI (percutaneous coronary intervention) is performed, the same method can allow assessment over time of the precise residual stenosis after stenting and verify the microvasculature status by evaluating coronary flow reserve.

## 1. Introduction

Wellens’ syndrome (WS) is a preinfarction stage of coronary artery disease. It was first reported in 1982 and consisted of a pattern of electrocardiographic ST/T-wave changes associated with critical proximal left anterior descending (LAD) artery stenosis in patients whose symptoms are consistent with unstable angina [1,2,3].

The pathophysiology of the syndrome is basically attributable to a sub-occlusive acute thrombotic complication of an atherosclerotic plaque in the proximal left anterior descending coronary artery (LAD). Several factors (smoking, oxidized LDL, environmental toxicants and chemicals, ionizing radiation, and ongoing infection, to name just a few) can destabilize the plaque and provoke an acute complication [4]. The new 2019 coronavirus (SARS-CoV-2) is primarily deemed to be a respiratory disease (COVID-19). However, several studies have shown a multitude of cardiac reverberations in the setting of COVID-19, including acute myocardial infarction (AMI), arrhythmias, myocarditis, left ventricular dysfunction and acute coronary events [5,6]. Notwithstanding such reports, a link between COVID and an acute coronary syndrome like Wellens’ syndrome has been reported only once before; in that circumstance, it appeared as a pseudo-WS [7].

Most importantly, a totally non-invasive, possibly bedside approach to confirm the status of the LAD in this syndrome would be very useful, as also in cases of percutaneous coronary intervention (PCI) to assess the stent patency and microcirculation status after the procedure without exposing the patient to further ionizing radiation. Unfortunately, coronary computed tomography is fraught with limitations. First of all, it is not a bedside procedure; secondly, and most importantly, it involves ionizing radiation exposure and a contrast medium burden that can add dangerously to the same burden incurred during coronary angiography [8,9]. Finally, the plaque is only morphologically assessed and so offers poor accuracy in estimating the severity of the phenomenon, as well as the risk of false positives [10,11].

Transthoracic enhanced Doppler echocardiography (E-Doppler TTE) can assess obstructive atherosclerosis (both mild and severe) by recording blood flow velocity in the left main coronary artery (LMCA) and the whole left anterior descending coronary artery (LAD) in a totally non-invasive way (no exposure to either ionizing radiation or iodinated contrast medium) [11,12,13] with a feasibility of almost 100%. This allows, in cases of stenosis, Doppler recordings of transtenotic blood flow velocity to be made. The epicardial conduit flow mapping is usually combined with coronary flow reserve assessment in the distal segment of the LAD [11,14]. It is the only approach that can combine direct epicardial coronary vessel evaluation with the assessment of coronary flow reserve in the same territory [11,15]. Its ample feasibility is due mainly to heart rate lowering and to the sensitive Doppler technology’s ability to identify a low-velocity, weak-intensity signal like coronary blood flow combined with new tomographic planes [13,16].

However, E-Doppler TTE has never been tested in an acute syndrome like WS.

We presented a clinical case observed at the Echo Lab of our institution. This is the first time that a COVID-19- triggered WS has been described and, at the same time, offers a first report of the role of E-Doppler TTE in this acute context before and after the PCI.

## 2. Clinical Case

**Figure 1:**
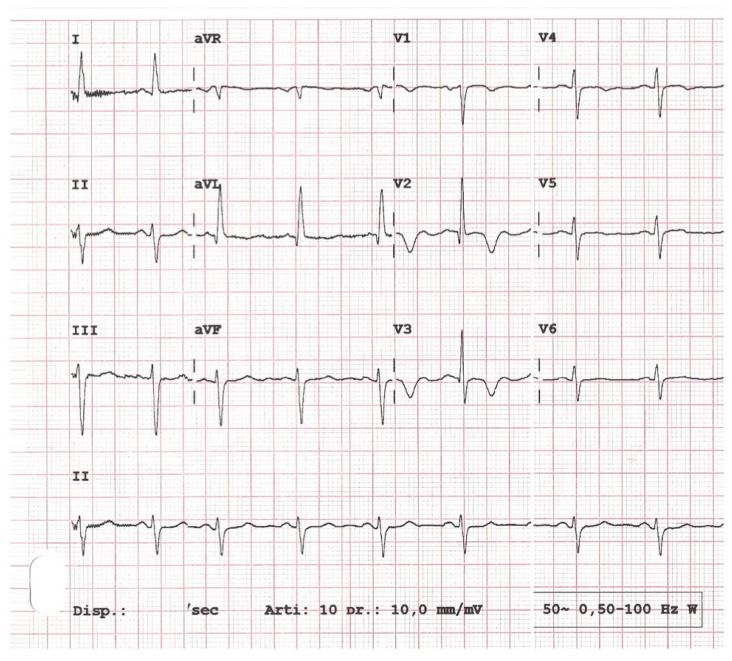
EKG at entry, showing deeply inverted T waves in V2–3 with no displaced ST segment. This can indicate prolonged septal ischemia followed by reperfusion, usually due to a complicated, severe plaque in the proximal LAD.

A 69-year-old man had started to complain 3 days before of typical chest pain, mainly after moderate effort but also during a brief episode at rest. He had never experienced such symptoms before. Past medical history was negative, except for the presence of systemic arterial hypertension due to metabolic syndrome, which was pharmacologically treated with sartans for at least 20 years, and of severe prostate hyperplasia, which was surgically treated 2 years before. Other cardiovascular risk factors were absent. Remarkably, a couple of weeks before, he had experienced first a sore throat, for about 3–4 days, with fever at 38.5°, followed by a cough with minimal production of sputum. This worsened progressively over the next 2–3 days, and he complained of fatigue. The symptoms did not alarm him since they resembled other aspecific episodes of upper respiratory infection suffered in the past, so he decided to stay at home and did not seek medical attention. In two weeks, he recovered, but given the COVID pandemic, he decided to undergo a nasopharyngeal swab for SARS-CoV-2 RT-PCR assay before returning to work. In the beginning, this was negative. After the onset of the new chest pain, he came to our clinical tertiary center. He was scheduled as an outpatient; a second nasopharyngeal swab the day before this cardiologic evaluation was again negative. Immediately after entering the echocardiography lab, he began complaining of typical, oppressive retrosternal chest discomfort and shortness of breath lasting a few minutes that spontaneously regressed. He appeared alert and oriented, and frankly overweight. Vital signs were normal: blood pressure was 130/70 mmHg; heart rate was 64 bpm; peripheral oxygen saturation was 99% in room air; a normal respiratory rate (15 b/m) was present; and there was no fever. Physicals showed no palpable apex beat, a normal jugular pulse with an X’ descent and a normal arterial pulse (normal rate of rising with normal amplitude), but auscultation revealed an intense IV heart sound. The EKG showed signs of reperfusion after prolonged ischemia in the LAD territory: a sinus rhythm with deeply inverted T waves in V2–3, and T-wave flattening in V4–V6 resembling Wellens’ EKG syndrome (Figure 1). He underwent general transthoracic echocardiography followed by enhanced coronary Doppler echocardiography.

**Figure 2:**
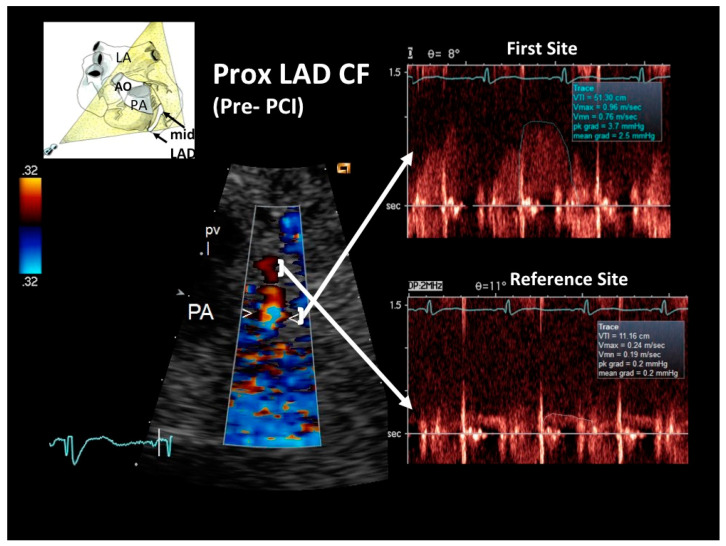
Blood flow Doppler recording by E-Doppler TTE in the proximal LAD, offering a modified short-axis view of the base of the heart in the acute phase. On the left, color flow in the proximal LAD shows a limited area of considerably accelerated BFV expressed by aliased signals (arrowheads); some flushing artifacts are also present since the heart rate was suboptimally reduced, but they do not disturb the proper signal interpretation. PW Doppler sampling (on the right) at the color-aliased signal confirms a critical increase of blood flow velocity compared to the distal reference area (upper and lower arrows connect the sampling color area to the corresponding PW Doppler tracing). Application of the continuity equation yields an 89% stenosis area. A cartoon indicating the plane orientation is shown on the upper left. The diastolic waves of the Doppler tracings are outlined in blue. LAD = left anterior descending coronary artery; PW = pulsed wave; CF = coronary flow; RVOT = right ventricular outflow tract; PV = pulmonary valve; PA = pulmonary artery; LA = left atrium.

General echocardiography revealed marked apical and anterior septal wall hypokinesia, with a globally preserved left ventricular systolic function but marked telediastolic restriction, as assessed by pulmonary venous flow Doppler recording. The latter showed a high peak and markedly prolonged duration of reversal of pulmonary venous flow [17]. E-Doppler TTE allowed a flow velocity Doppler recording to be made in the LMCA and in the entire LAD. Findings were markedly abnormal, recording a locally accelerated and turbulent flow at the end of the proximal LAD tract, as revealed by color-guided PW Doppler recording indicating significant stenosis (Figure 2). By applying the continuity equation, which has been amply validated with this approach [12,13,18], the stenosis was quantified as severe (89% of area reduction). Coronary flow reserve (CFR) was not measured due to the critical setting.

**Figure 3:**
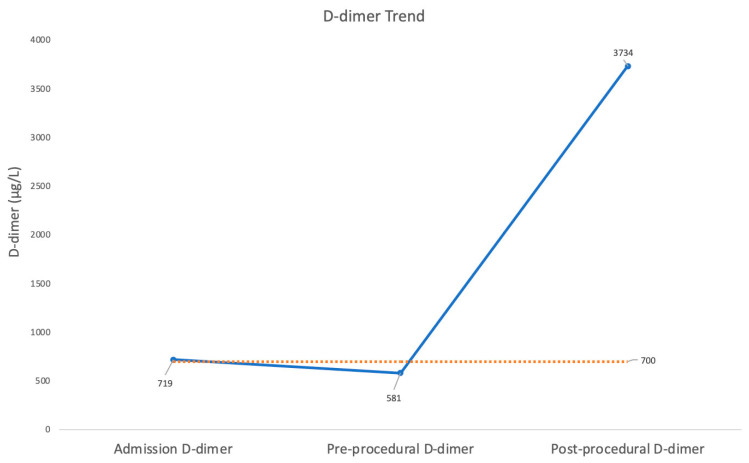
Time course of D-dimer. The D-dimer concentration was plotted on admission, immediately before PCI and after PCI. The D-dimer level was mildly elevated at admission; it tended to decrease immediately before PCI (24 h after admission) but increased considerably immediately after PCI. PCI = percutaneous coronary intervention.

On the basis of this evidence, hospital admission for urgent PCI was decided upon. Following the COVID-19 pandemic protocol, before admission, the patient was checked for COVID-19 infection by SARS-CoV-2 reverse transcription-polymerase chain reaction (RT-PCR) from a nasopharyngeal swab. This time the test results were positive, tested twice, 6 h apart. The patient was sent to the intensive care unit (ICU) for COVID-19 patients. There, the repeat ECG was unchanged, and the serum level of cardiac high-sensitivity troponin T (I assay) was at 1843.5 pg/mL (normal value < 0.014 pg/mL). In addition, he showed a mild increase of D-dimer (Figure 3).

**Figure 4:**
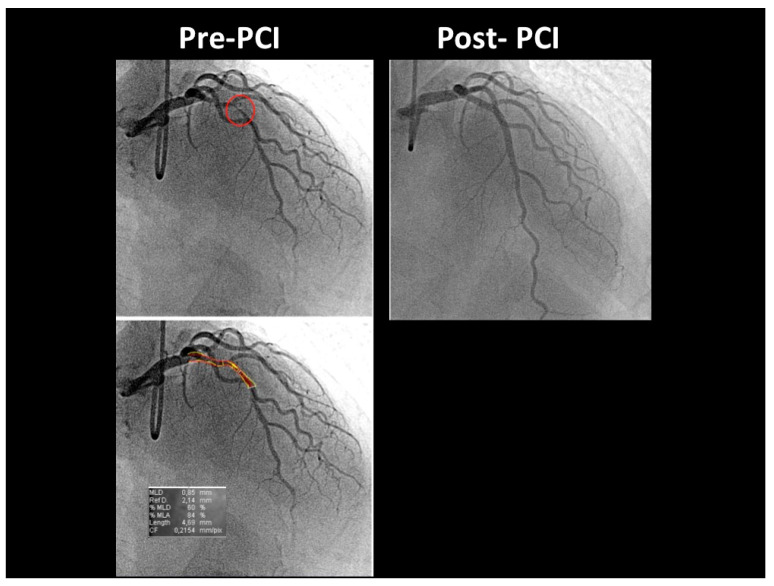
Coronary angiography before and after PCI. Before PCI (**top left**), coronary angiography (CRA 33° RAO 27°) showed a long, significant critical stenosis (red circle) of the proximal LAD; the (**bottom left**) image shows the QCA of the same pre-PCI image, demonstrating an 86% area reduction; after PCI, coronary angiography (**top right**) (CRA 35° RAO 14°) shows the optimal final result, with no residual stenosis. PCI = percutaneous transluminal coronary angiography; QCA = quantitative coronary angiography; CRA = cranial; RAO = right anterior oblique view; PCI = percutaneous coronary intervention.

**Figure 5:**
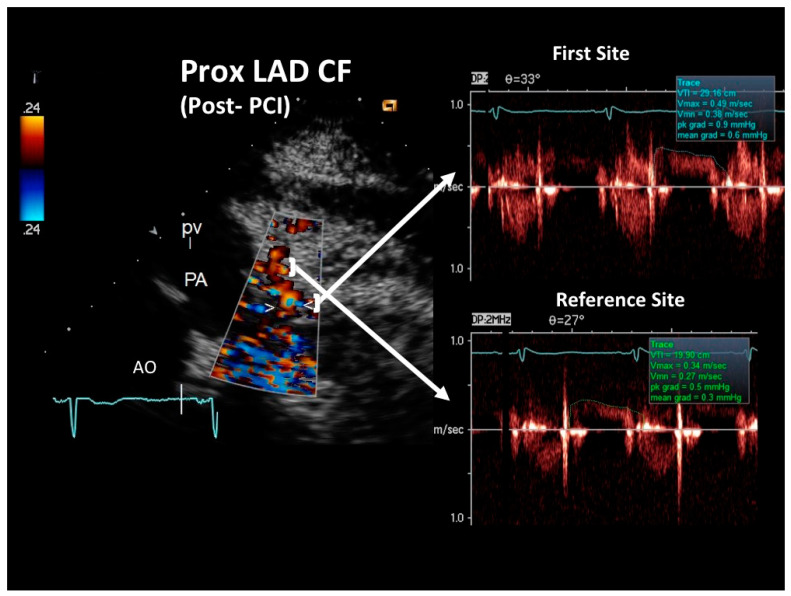
Blood flow Doppler recording by E-Doppler TTE in the proximal LAD after stenting in the follow-up (same format as in the acute phase). Maximal velocity had dropped to 49 cm/s, yielding a residual stenosis area of 25% according to the continuity equation. The diastolic waves of the Doppler tracings are outlined in green, and the maximal velocity and time velocity integral are reported. Abbreviations as in Figure 2.

**Figure 6:**
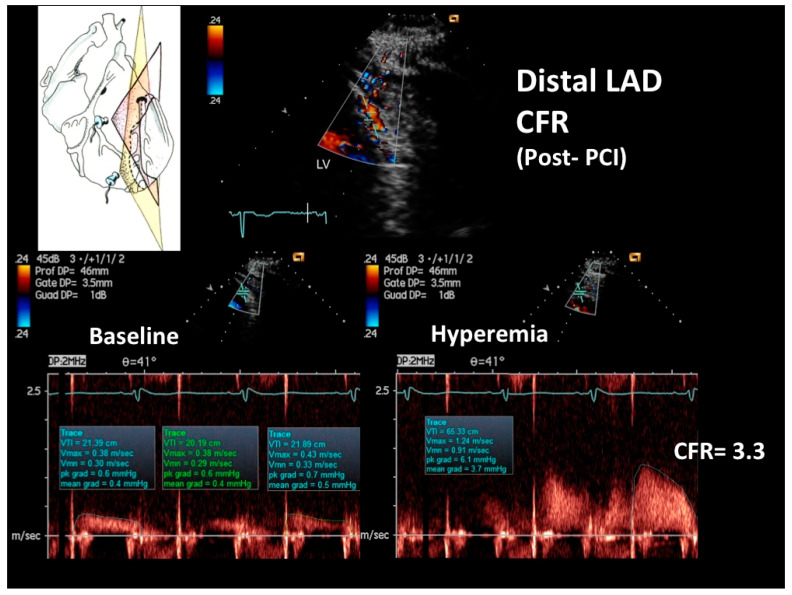
The CFR in the distal LAD was assessed by E-Doppler TTE in the follow-up after PCI. At the top, color flow in the distal LAD (in red); on the left, a cartoon of the tomographic plane orientation to obtain the distal LAD in sonification. At the bottom, PW spectral tracing of the blood flow velocity in the distal LAD at baseline (**left**) and at maximal adenosine-induced hyperemia (**right**); note the prevalent diastolic BF velocity, a peculiarity of coronary flow. The CFR (peak hyperemic diastolic velocity/peak resting diastolic velocity) is above 3, indicating no significant stenosis and a normal microcirculatory function. CFR = coronary flow reserve; PCI = percutaneous coronary intervention; PW = pulsed-wave Doppler; BF=blood flow.

At the COVID intensive care unit, he was admitted to the catheterization laboratory, where he received immediate intravenous therapy, 5000 IU heparin sodium, 250 mg lysine acetylsalicylate and cangrelor bolus followed by continuous infusion (according to body weight). Coronary angiography showed sub-occlusive stenosis (lumen narrowing = 86%) of the proximal LAD (Figure 4), which was treated with balloon angioplasty followed by drug-eluting stent placement (Resolute Onyx 2.75 × 34 mm). Following the intervention, 0% residual stenosis was recorded, with an optimal residual flow and no dissection present (Figure 4). A total of 2 hours post-procedure, 180 mg oral ticagrelor (after the suspension of cangrelor) was administered, followed, from the following day, by 100 mg oral aspirin daily, 90 mg oral ticagrelor bis in die (as part of a dual antiplatelet regimen) and goal-directed medical therapy with a beta-blocker and highly intense statin therapy [19]. The ECG following PCI displayed persistent pseudo-normalization of T waves in the precordial leads; cardiac high-sensitivity troponin T (III assay) increased considerably to 3734 μg/L. Patient recovery was event-free, and he was transferred on the fourth day to the internal medicine department, pending the negativization of the SARS-CoV-2 molecular assay. He was discharged on the 7th day on dual antiplatelet therapy but not on anticoagulation therapy since his COVID infection had no thromboembolic complications [20]. After two months, he was re-evaluated by E-Doppler TTE, this time adding CFR assessment. The transtenotic velocity had dropped almost completely, indicating a patent stent (minimal residual stenosis at 26% of area reduction according to the continuity equation) (Figure 5), and the CFR was also normal (=3.3) (Figure 6), confirming the stent patency and complete recovery of microcirculatory function in the LAD myocardial territory.

## 3. Discussion

This case underlines that WS can be triggered by COVID-19 infection. Most importantly, we show for the first time that this can be confirmed at the bedside by means of E-Doppler TTE, a totally non-invasive approach that allows direct detection and also an assessment of the severity of stenosis of the LAD.

Acute coronary syndrome has already been linked to COVID-19 infection [7]. This is the second report to link WS to a COVID-19 infection. The previous case, however, appeared as a pseudo-WS since that patient had no chest pain but had COVID pneumonia with global ventricular dysfunction and possibly secondary cardiac injury. At coronary angiography, only a distal circumflex coronary artery showed critical stenosis, with no involvement of the LAD [7].

COVID-19 infection is an etiologic cofactor for WS. On the basis of the criteria set out to assess causality 80 years ago by Sir Bradford, professor of medical statistics [21], viral respiratory infections have been accepted as an etiological factor in triggering acute coronary syndrome [22]. Our patient with COVID confirms this point. In fact, several factors support the concept that COVID infection induced the WS in our patient. The first element was the temporal relation (WS two weeks after the respiratory syndrome); in fact, the risk of coronary complications is maximal in the first few days after the onset of the respiratory syndrome and falls over time but can remain higher at three months. Secondly, the infection has been reported to trigger a coronary complication in several populations (consistency principle). Thirdly, the patient was overweight with insulin resistance, a serious risk factor for coronary complications after acute infection (coherence principle). Finally, it is plausible that the molecular mechanisms causing the syndrome could be induced by COVID because all infections are a risk factor for acute coronary syndrome [23], especially if the infection is prolonged (albeit intermittent), as in our case. In fact, infections destabilize plaque through the combination of humoral mechanisms (both systemic and local) with increasing hemodynamic forces that mechanically disrupt the plaque [24]. One humoral mechanism that is critical in COVID infection causing ACS is thrombophilic status, which can increase thrombus formation not only in the venous system but also in the arterial conduit [22]. In COVID patients, floating thrombi have been described in the aorta and in the femoral arteries, in this last case causing particularly severe lower limb ischemia [22]. COVID-19 infection, based on the cytokine storm, can dramatically unbalance the coagulation system, favoring thrombus formation. Our patient had a mild D-dimer increase when we saw him at the beginning, confirming the role of a hypercoagulable state in causing arterial thrombus formation (Figure 3).

Technical comments on E-Doppler TTE. Echocardiographic equipment characteristics and settings for these examinations have been previously reported [13]. Briefly, the enhancement of this echo approach consists first of using a very sensitive Doppler technology based on power Doppler (convergent color Doppler mode) suitable to detect a low-velocity, weak-intensity signal, such as the coronary flow signal. Second, the coronary insonification time is prolonged by pharmacologically reducing the heart rate below 60 b/min. At this heart rate, the diastolic time is disproportionally lengthened; thus, longer insonification times are obtained in that part of the cardiac cycle where the coronaries are still and clutter artifacts from tissue are absent (in our case the HR was suboptimally lowered at around 64 b/m; this didn’t hamper blood flow Doppler recording but reduced Doppler quality a little). This evaluation is performed using an Acuson Sequoia ultrasound unit (C256 Echocardiography System, Siemens Healthcare, Erlangen, Germany) and a broadband transducer (3V2c). Other pieces of equipment with a beamformer array and sensitive Doppler module work equally well, such as the GE Vivid 7 equipment. B-mode images are obtained in tissue harmonics (incident waves in fundamental at 2 MHz and receiving in the second harmonic at 4 MHz). The color Doppler signal is attained in convergent color Doppler mode at 2.5 MHz transmission frequency, while spectral Doppler is performed in fundamental mode at 2.5 MHz. The color-coded Doppler setting was adjusted to maximize scanning sensitivity (the pulse repetition frequency is set at 16 cm/s with minor modulation in special cases, maximizing the sample volumes of the color scan lines) without significantly reducing the frame rate (the color box size is reduced to remain in keeping with a frame rate of >30 Hz).

**Figure 7:**
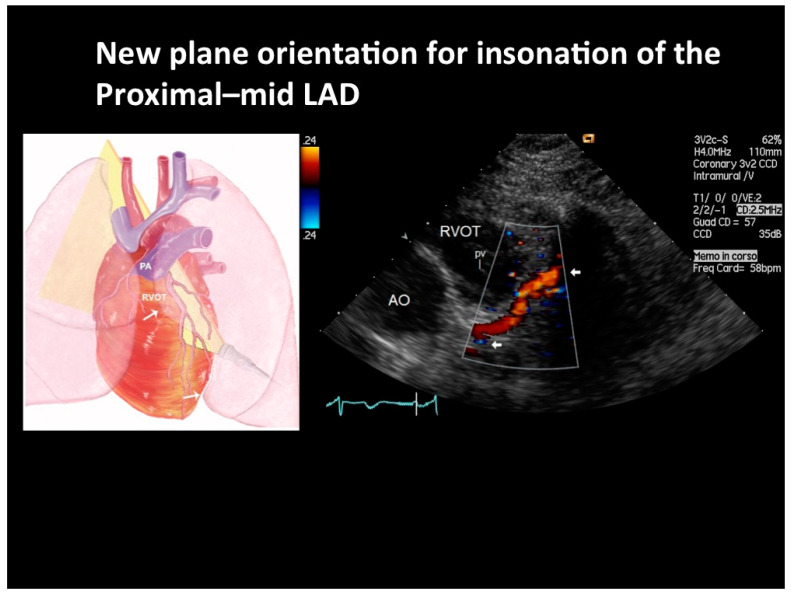
New tomographic plane orientation for LAD insonation. Color flow in the proximal and in the very short segment of the mid LAD is shown (indicated by arrows). Ultrasound plane orientation is depicted in the cartoon on the left side; see text for explanations. RVOT = right ventricular outflow tract; PV = pulmonary valve; AO = aorta.

Last but not least, technical advances of this enhanced method are represented by new tomographic planes that add up to those first described [12]. These new tomographic plane orientations have been described elsewhere [13]. Briefly, the color flow of the proximal and mid-LAD was substantially improved first by placing the patient in extreme lateral decubitus in order to displace the lungs laterally and uncover the heart as much as possible, and then exploiting the cardiac notch of the left lung by translating the transducer as far as possible laterally on the left hemithorax while keeping optimal heart insonification (Figure 7) (Supplementary Materials Video S1). In this way, the LAD is brought, with a good theta angle, in the center of the sector, where the output energy of the transmitted ultrasound is maximal (Figure 7). The probe is then minimally angled medially and cranially toward the left coronary fossa and the interventricular sulcus. In this way, a longer tract of the artery (mainly the conjunction of the retropulmonary and the proximal interventricular portion) is generally insonified on the same plane with a narrow theta angle (Figure 7), and the diagonal branches are also almost regularly insonified. In addition, apical views can also be attempted in very difficult patients with retrosternal heart and/or poor parasternal windows [13]. Starting from a four-chamber view, the probe is angled anteriorly in order to bring the aorta root (oblique slice) into view. A close search for the flow in the left coronary fossa, adjacent to the left side of the aortic root, is attempted by moving the transducer slightly back and forth in order to record blood flow in the LMCA and proximal LAD.

The importance of E-Doppler TTE in WS is highlighted. This approach allows Doppler recording of blood flow velocity in the LMCA and in the entire LAD to be made, and the acceleration of blood flow at the stenosis site (normal physics effect in order to maintain blood flow constant [25]) can be appropriately measured. This accelerated velocity at the stenosis site, along with a reference velocity attained immediately distally or proximally to the stenosis acceleration, enables us to use the continuity equation in order to quantitate the stenosis severity [12,13,18]. In addition, E-Doppler TTE allows the assessment of coronary flow reserve (CFR) in the distal LAD, which is a post-stenotic CFR assessment [14,26,27]. This evaluation, as opposed to the pre-stenotic CFR, where the maximal flow can be pseudo-normalized by the hyperemic increase in flow in the branches stemming off the LAD between the sample site and the stenosis, precisely reflects the dumping effect of the obstructing plaque [28]. Therefore, the ischemic potential of the plaque can be accurately inferred [14,27,29]. However, a low CFR can also be due to microcirculatory dysfunction. E-Doppler TTE can provide a way to differentiate between these 2 conditions of low CFR: a critical accelerated blood flow (≥92 cm/s as validated versus fractional flow reserve), being an excellent marker of critical stenosis [30], indicates a low CFR due to stenosis and not a microcirculation dysfunction, as recently preliminarily demonstrated for the first time [15]. Finally, E-Doppler TTE evaluation does not expose the patient to cancerogenic ionizing radiation or nephrotoxic iodinated contrast medium [8].

E-Doppler TTE has largely been tested in chronic patients but has not yet been validated in the acute setting like WS. It appears to be an ideal tool in the presence of an EKG indicating WS. In fact, Wellens’ syndrome is a high-risk situation due to the acute complication of a plaque in the proximal LAD, with ensuing sub-occlusion at this level of the vessel. The risk of further complication with anterior wall myocardial infarction, leading to significant left ventricular dysfunction and/or death, is high [2]. It is, therefore, clear that in cases of suspected WS based on clinical grounds, a bedside, non-invasive approach like E-Doppler TTE that allows rapid detection, as well as a LAD stenosis location and severity assessment, is crucial.

In fact, the exclusion of stenosis excludes the syndrome that can be mimicked by pseudo-Wellens’ syndrome (cases with the same EKG pattern of repolarization abnormality as true WS but with no coronary subocclusion). Such pseudo-Wellens’ syndromes could occur after cocaine use [31], marijuana use [32], myocardial bridging [33], pulmonary embolism [34], and Taktzubo syndrome [35]. On the other hand, when stenosis is present, the possibility of gauging the stenosis severity and following up the stenosis at the bedside in a totally non-invasive way, and most importantly, with no radiation and contrast exposure, may allow other, more conservative therapeutic strategies to be implemented. In our case, the stenosis was severe but at the same time guaranteed a certain perfusion of the muscle. Therefore, the urgent therapeutic step towards coronary angiography and eventually PCI may not have been so compelling. An aggressive antithrombotic therapy (heparin bolus 100 U/kg IV followed by a maintenance infusion of 18 mcrg/kg/min plus antiaggregation) along with therapy contrasting the paradox spasm (nitrates, and if necessary alpha-beta blockers) in ICU monitoring for 24–48 h, with a revaluation of the severity of the stenosis by E-Doppler TTE, could be a further option. With the passage of time, the infection-induced cytokine storm might subside and eventually, the thrombus component of the obstruction also aided by the anticoagulation therapy (that mainly contrasts further clot formation) could be to a level no longer warranting PCI. Further studies are needed to confirm the validity of this strategy.

However, in our case, the COVID infection hampered such strategies owing to the difficulty and operator risk of repeated bedside monitoring.

As expected, the reference distal flow velocity was low (Figure 2) and mildly increased after PCI (Figure 5). A long severe stenosis can reduce basal flow and flow velocity distally to the obstruction. This, however, does not hamper stenosis severity assessment by the continuity equation as previously demonstrated. Even if the stenosis is very severe (>85% diameter narrowing) and there is a reduction at rest of the flow distally to the stenosis, as demonstrated by the landmark Gould study [36], the flow at the stenosis and in the post-pre-stenotic segments must be the same. This arises on the basis of the principle of continuity of flow, which is a corollary of the law of conservation of mass, which states that the flow in any portion of a non-branching tube is equal: what comes in must come out [25]. Problems can arise in cases of collaterals between the stenosis and the sampling site, since flow could be diverged to the collaterals and so create flow differences between the sampling site and the stenosis, causing inaccurate estimation of the % lumen narrowing with the continuity equation [37,38]. However, as demonstrated in a recent in vitro study [38], since there is a certain pressure drop in the post-stenotic segment, such a divergent flow to collaterals is much less important in cases of sampling distally to the stenosis than proximally, owing to a reduced driving pressure toward collaterals in the distal compared to the proximal segments. Therefore, the authors of that paper suggest that as a general rule, it is better to sample the reference distally (as in our case) rather than proximally, owing to the risk of such collaterals.

The good reproducibility of the method, as recently reported, allows a reliable follow-up of the transtenotic velocity [13]. In fact, the repeatability coefficient of % CSA (cross-sectional area) stenosis is 13.8% (95% CI: 10–22%), meaning that a % CSA stenosis variation exceeding this value is a real variation and not a random noise of the technique. In our case, when assessing by E-Doppler TTE, the variation of % CSA was much larger than the repeatability coefficient of % CSA (from 89% to 25% of CSA), pointing out the effective reduction of the stenosis by the PCI procedure, as confirmed by post-PCI coronary angiography. The residual intra-stent acceleration contrasting the absence of stenosis by angiography is a frequent finding, as already pointed out using the intravascular Doppler flow wire technique [39] and confirmed by our method [12].

Limitations of E-Doppler TTE in the acute setting. HR reduction is mandatory in order to improve the signal-noise ratio of the coronary blood flow backscatter. This can be a problem in an acute setting since, in cases of elevated HR (that is the rule in the ICU), the exam cannot be performed until a proper reduction of HR (around 60 b/m but better <60 b/m) has been obtained [13,16]. However, most of the time, a proper HR reduction, even in the acute setting, can be achieved with full-dosage beta-blockers (metoprolol 5 mg EV in 5 min, repeated, if necessary, twice at 10 min intervals until a target HR has been achieved, obviously under strict blood pressure control in order to avoid hypotension) [13]. In cases where heart failure is present, first, the patient has to be stabilized (so as to reduce the sympathetic drive), and then the exam is performed. In our case, a simple mini-dose of beta-blockers (PO) induced a decrease in HR that, despite being sub-optimal (60–65 b/m), allowed blood flow Doppler recording. The more difficult the chest situation, the lower the HR should be.

Coronary flow reserve (CFR) assessment in the distal LAD, the natural completion of this exam in order to confirm the severity of the hemodynamic pressure drop at the level of the plaque, cannot be performed in patients with an ongoing ischemic state since adenosine, through the stealing phenomenon, might induce further muscle damage and eventually acute arrhythmias. In our case, however, a low CFR (<2.0) was more than expected, owing to the association with a ≥80–90% stenosis, that per se induces a pressure drop in the post-stenotic segment, significantly reducing the reserve [15], along with a reduced vasodilatory capability of the stunned microvasculature owing to ischemic damage [40]. However, the CFR assessment was performed in the follow-up and, thanks to the combination with the transtenotic velocity assessment, offered precious insights, confirming both the patency of the stent and a good microcirculatory recovery after acute post-ischemic myocardial stunning.

## 4. Conclusions

COVID-19 infection can trigger a true WS by destabilizing even an isolated plaque, creating a procoagulant state and eventually more biomechanical stress exerted on the plaque. A diagnostic method like E-Doppler TTE features high feasibility and extremely high diagnostic accuracy for the assessment of LAD stenosis and does not expose patients to harmful agents (neither ionizing radiation nor contrast medium). It is, therefore, repeatable and an ideal tool for clinical decision making in cases where this syndrome is suspected.

## Data Availability

Not applicable.

## Supplementary Materials

The following supporting information can be downloaded at: https://www.mdpi.com/article/10.3390/diagnostics12040804/s1, Video S1.

## References

[1] De Zwaan C., Bär F.W., Janssen J.H., Cheriex E.C., Dassen W.R., Brugada P., Penn O.C.K.M., Wellens H.J.J. (1989). Angiographic and clinical characteristics of patients with unstable angina showing an ECG pattern indicating critical narrowing of the proximal LAD coronary artery. Am. Heart J..

[2] Rhinehardt J., Brady W.J., Perron A.D., Mattu A. (2002). Electrocardiographic manifestations of Wellens’ syndrome. Am. J. Emerg. Med..

[3] Kannan L., Figueredo V.M. (2015). Images in clinical medicine. Wellens’ syndrome. N. Engl. J. Med..

[4] Caiati C. (2019). Contrast-Enhanced Ultrasound Reveals That Lipoprotein Apheresis Improves Myocardial But Not Skeletal Muscle Perfusion. JACC Cardiovasc. Imaging.

[5] Shi S., Qin M., Shen B., Cai Y., Liu T., Yang F., Gong W., Liu X., Liang J., Zhao Q. (2020). Association of Cardiac Injury With Mortality in Hospitalized Patients With COVID-19 in Wuhan, China. JAMA Cardiol..

[6] Wang D., Hu B., Hu C., Zhu F., Liu X., Zhang J., Wang B., Xiang H., Cheng Z., Xiong Y. (2020). Clinical Characteristics of 138 Hospitalized Patients With 2019 Novel Coronavirus-Infected Pneumonia in Wuhan, China. JAMA.

[7] Elkholy K.O., Mirashi E., Malyshev Y., Charles G., Sahni S. (2021). Wellens’ Syndrome in the Setting of the 2019 Novel Coronavirus (COVID-19). Cureus.

[8] Nguyen P.K., Lee W.H., Li Y.F., Hong W.X., Hu S., Chan C., Liang G., Nguyen I., Ong S.-G., Churko J. (2015). Assessment of the Radiation Effects of Cardiac CT Angiography Using Protein and Genetic Biomarkers. JACC Cardiovasc. Imaging.

[9] Faucon A.L., Bobrie G., Clement O. (2019). Nephrotoxicity of iodinated contrast media: From pathophysiology to prevention strategies. Eur. J. Radiol..

[10] Nissen S.E. (2008). Limitations of computed tomography coronary angiography. J. Am. Coll. Cardiol..

[11] Caiati C., Scardapane A., Iacovelli F., Pollice P., Achille T.I., Favale S., Lepera M.E. (2021). Coronary Flow and Reserve by Enhanced Transthoracic Doppler Trumps Coronary Anatomy by Computed Tomography in Assessing Coronary Artery Stenosis. Diagnostics.

[12] Caiati C., Zedda N., Cadeddu M., Chen L., Montaldo C., Iliceto S., Lepera M.E., Favale S. (2009). Detection, location, and severity assessment of left anterior descending coronary artery stenoses by means of contrast-enhanced transthoracic harmonic echo Doppler. Eur. Heart J..

[13] Caiati C., Lepera M.E., Pollice P., Iacovelli F., Favale S. (2020). A new noninvasive method for assessing mild coronary atherosclerosis: Transthoracic convergent color Doppler after heart rate reduction. Validation vs. intracoronary ultrasound. Coron. Artery Dis..

[14] Caiati C., Montaldo C., Zedda N., Bina A., Iliceto S. (1999). New noninvasive method for coronary flow reserve assessment—Contrast-enhanced transthoracic second harmonic echo Doppler. Circulation.

[15] Caiati C., Lepera M., Pollice P., Favale S. (2020). Non Invasive Detection of Accelerated Stenotic Flow in The Entire Left Anterior Descending Coronary Artery Provides Insight into the Causes of Impaired Coronary Flow Reserve: A Study Conducted with Enhanced Transthoracic Convergent Color Doppler Echocardiography. J. Am. Coll. Cardiol..

[16] Caiati C., Lepera M., Pollice P., Favale S. (2020). Feasibility of a New Non Invasive Method for the Evaluation of Coronary Blood Flow in Coronaries: Transthoracic Convergent Color Doppler Mode along with Pharmacologically Induced Heart Rate Lowering. J. Am. Coll. Cardiol..

[17] Caiati C., Argentiero A., Forleo C., Favale S., Lepera M.E. (2021). Predictors of Exercise Capacity in Dilated Cardiomyopathy with Focus on Pulmonary Venous Flow Recorded with Transesophageal Eco-Doppler. J. Clin. Med..

[18] Caiati C., Lepera M.E., Santoro D., Grande D., Iacovelli F., Tarantino N., Tito A., Lacitignola I., Basile M., Masi F. (2013). Assessment of the Severity of Left Anterior Descending Coronary Artery Stenoses Using Transthoracic Enhanced Doppler Echocardiography in Convergent Color Doppler Mode. Validation of a Method Based on the Continuity Equation. J. Am. Coll. Cardiol..

[19] Angeli F., Reboldi G., Mazzotta G., Garofoli M., Cerasa M.F., Verdecchia P. (2012). Statins in acute coronary syndrome: Very early initiation and benefits. Ther. Adv. Cardiovasc. Dis..

[20] Chandra A., Chakraborty U., Ghosh S., Dasgupta S. (2021). Anticoagulation in COVID-19: Current concepts and controversies. Postgrad. Med. J..

[21] Hill A.B. (1965). The Environment and Disease: Association or Causation?. Proc. R Soc. Med..

[22] Corrales-Medina V.F., Madjid M., Musher D.M. (2010). Role of acute infection in triggering acute coronary syndromes. Lancet Infect. Dis..

[23] Nguyen J.L., Yang W., Ito K., Matte T.D., Shaman J., Kinney P.L. (2016). Seasonal Influenza Infections and Cardiovascular Disease Mortality. JAMA Cardiol..

[24] Biasucci L.M., Leo M., De Maria G.L. (2008). Local and systemic mechanisms of plaque rupture. Angiology.

[25] Yoganathan A.P., Cape E.G., Sung H.W., Williams F.P., Jimoh A. (1988). Review of hydrodynamic principles for the cardiologist: Applications to the study of blood flow and jets by imaging techniques. J. Am. Coll. Cardiol..

[26] Caiati C., Montaldo C., Zedda N., Montisci R., Ruscazio V., Lai G., Cadeddu M., Meloni L., Iliceto S. (1999). Validation of a new noninvasive, method (contrast-enhanced transthoracic second harmonic echo Doppler) for the evaluation of coronary flow reserve—Comparison with intracoronary Doppler flow wire. J. Am. Coll. Cardiol..

[27] Caiati C., Zedda N., Montaldo C., Montisci R., Iliceto S. (1999). Contrast-enhanced transthoracic second harmonic echo Doppler with adenosine: A noninvasive, rapid and effective method for coronary flow reserve assessment. J. Am. Coll. Cardiol..

[28] Donohue T.J., Kern M.J., Aguirre F.V., Bach R.G., Wolford T., Bell C.A., Segal J. (1993). Assessing the hemodynamic significance of coronary artery stenoses: Analysis of translesional pressure-flow velocity relations in patients. J. Am. Coll. Cardiol..

[29] Caiati C., Becher H., Burns P.N. (2000). Coronary Flow Reserve. Handbook of Contrast Echocardiography.

[30] Caiati C., Lepera M.E., Santoro D., Grande D., Tito A., Marolla P., Stufano M., Meliota G., Iacovelli F., Masi F. (2014). Physiologic Significance Assessment of Intermediate Severity Coronary Lesions by Transthoracic Enhanced Doppler Echocardiography in Convergent Color Doppler Mode. Validation versus Fractional Flow Reserve. J. Am. Coll. Cardiol..

[31] Dhawan S.S. (2008). Pseudo-Wellens’ syndrome after crack cocaine use. Can. J. Cardiol..

[32] Co M.L.F., Das A., Okwuosa T. (2017). Pseudo-Wellens syndrome after heavy marijuana use. Cleve. Clin. J. Med..

[33] Kaplanis I., Michas G., Arapi S., Thomopoulos T., Stougiannos P., Trikas A. (2017). Myocardial bridge as a cause of pseudo-Wellens’ syndrome. Hell. J. Cardiol..

[34] Sedhai Y.R., Basnyat S., Bhattacharya P.T. (2018). Pseudo-Wellens’ syndrome in pulmonary embolism. BMJ Case Rep..

[35] Taylor R.S., Skjerli L., Ashurst J. (2017). Takotsubo Cardiomyopathy Presenting as Wellens’ Syndrome. Clin. Pract. Cases Emerg. Med..

[36] Gould K.L., Lipscomb K., Hamilton G.W. (1974). Physiologic basis for assessing critical coronary stenosis. Instantaneous flow response and regional distribution during coronary hyperemia as measures of coronary flow reserve. Am. J. Cardiol..

[37] Johnson E.L., Yock P.G., Hargrave V.K., Srebro J.P., Manubens S.M., Seitz W., Ports T.A. (1989). Assessment of severity of coronary stenoses using a Doppler catheter. Validation of a method based on the continuity equation. Circulation.

[38] Hozumi T., Yoshikawa J., Yoshida K., Akasaka T. (1995). Estimation of severity of stenosis with a Doppler guide wire in the experimental models. J. Am. Soc. Echocardiogr. Off. Publ. Am. Soc. Echocardiogr..

[39] Albertal M., Regar E., Van Langenhove G., Carlier S.G., Piek J.J., De Bruyne B., Di Mario C., Foley D., Kozuma K., Costaet M.A. (2002). Value of coronary stenotic flow velocity acceleration in prediction of angiographic restenosis following balloon angioplasty. Eur. Heart J..

[40] Rigo F., Sicari R., Citro R., Ossena G., Buja P., Picano E. (2009). Diffuse, marked, reversible impairment in coronary microcirculation in stress cardiomyopathy: A Doppler transthoracic echo study. Ann. Med..

